# Seroprevalence and “Knowledge, Attitudes and Practices” (KAPs) survey of endemic ovine brucellosis in Egypt

**DOI:** 10.1186/s13028-015-0183-2

**Published:** 2016-01-07

**Authors:** Yamen Hegazy, Walid Elmonir, Nour Hosny Abdel-Hamid, Essam Mohamed Elbauomy

**Affiliations:** 1 Animal Medicine Department, Faculty of Veterinary Medicine, Kafrelsheikh University, Kafrelsheikh, 33516 Egypt; 2 Hygiene and Preventive Medicine (Zoonoses) Department, Faculty of Veterinary Medicine, Kafrelsheikh University, Kafrelsheikh, 33516 Egypt; 3 Brucellosis Research Department, Animal Health Research Institute, Nadi El-Seid Street, Dokki, Giza, 12618 Egypt

**Keywords:** Sheep, Brucellosis, Seroprevalence, Risk factors, Shepherds’ KAPs, Control, Egypt

## Abstract

**Background:**

Between February and July 2014, a cross-sectional study to estimate the seroprevalence of brucellosis in sheep in the Kafrelsheikh district of Egypt was carried out, together with a survey of knowledge, attitudes and practices (KAPs) among local shepherds. A total of 273 serum samples were collected from 28 sheep flocks in 10 villages within the study area. These samples were analysed by the Rose Bengal Plate test (RBPT) test, with all positive samples being confirmed by complement fixation test (CFT).

**Results:**

True seroprevalence was 20 % (95 % CI 15.3–24.7 %) with the prevalence of villages with at least one seropositive sheep estimated at 95.5 % (95 % CI 92.2–100 %); village flock seroprevalence ranged from 0 to 46.8 %. Results of the KAPs survey demonstrated that despite good knowledge regarding brucellosis being potentially present within their flocks, shepherds lacked knowledge regarding routes of livestock to humans disease transmission and the symptoms of brucellosis in humans. This lack of knowledge regarding disease transmission resulted in high-risk practices being widespread—practices such as assisting parturition without protective measures, throwing aborted material into water canals and a reluctance to remove animals that had aborted from the flock.

**Conclusions:**

This study proposes potential measures to reduce seroprevalence of brucellosis in sheep and reduce public health risks from brucellosis such as culling aborted livestock and educational campaigns among shepherds regarding disease risks and modes of transmission.

**Electronic supplementary material:**

The online version of this article (doi:10.1186/s13028-015-0183-2) contains supplementary material, which is available to authorized users.

## Background

Brucellosis is a major zoonosis affecting public health and economy of many nations throughout the world, particularly in the Middle East, Mediterranean region, Central Asia and Latin America, where insufficient national control programmes has resulted in high endemicity [1, 2]. Brucellosis has however been eradicated from Japan, Canada, Australia, New Zealand and many countries in Northern and Central Europe [3]

Human brucellosis causes acute febrile illness with chronic hepatomegaly, splenomegaly and arthritis and is classified as a risk group III disease due to its ease of airborne transmission [4, 5]. Due to its debilitating nature, the disease has a major economic impact on patients, reducing their ability to work or support a family. The highest recorded incidences of human brucellosis are found in Central Asia and the Middle East [6].

In Egypt, the prevalence of human brucellosis was recently reported to be as high as 8 % in high-risk populations [7]. However, the true incidence of human brucellosis is not easy to known as many patients seek medical treatment in private clinics and not all of these cases are reported to the public health authorities. For instance, Jennings et al. [8] found that in Fayoum governorate, Egypt, hospital-based surveillance identified less than 6 % of the actual human brucellosis cases. Brucellosis in humans is strongly linked to contact with infected animals [9]. Therefore, farmers, shepherds, abattoir workers and veterinarians are considered as being the highest occupational risk groups [10].

In endemic areas, livestock brucellosis has a severe economic impact through lost productivity due to decreased milk production, abortions and infertility [2]. The high cost of brucellosis surveillance and control programmes is also an economic burden on low-income countries, along with associated impediments to trade [11].

Recent studies carried out in Egypt, particularly those in the area of this study, reported brucellosis to be endemic with high seroprevalence (12.2 % in sheep, 11.3 % in goats and 11 % in cattle) [9, 12]. The national control programme’s effectiveness in reducing this prevalence is questionable with brucellosis is still being present in all governorates of Egypt and up to 15 % of all livestock (cattle, buffaloes, sheep and goats) expected to be seropositive in some regions [13–15]. The national control programme was launched in 1985 and consisted of six-monthly serological testing of all female ruminants, with all seropositive livestock slaughtered and compensation provided for owners, together with voluntary vaccination of young female ruminants with either the S19 vaccine for cattle or Rev1 vaccine for sheep and goats [3].

Many factors have however reduced the programme’s effectiveness, such as; (1) a lack of reliable information on brucellosis seroprevalence in sheep (2) a lack of adequate communication between the public health authorities, veterinarians and stakeholders, (3) inadequate funding of surveillance and reporting systems, (4) the free movement of small ruminants between the various governorates in Egypt [13, 16].

The aim of this study was to estimate the flock-level seroprevalence of brucellosis among sheep in the Kafrelsheikh district of Egypt and describe the knowledge, attitudes and practices (KAPs) of shepherds, regarding brucellosis, in this district.

## Methods

In Egypt, small ruminants are raised mainly either as separate flocks (i.e. either sheep or goats) or as mixed flocks. They are kept in flocks managed by shepherds or by small-scale farmers, who work in growing crops and own small numbers of household reared animals for assistance in farming and for the use of their milk or meat [17, 18]. One shepherd would often keep sheep and goats from a number of different owners; as a result animals from different households are part of the same flock for grazing and breeding during most of the year. Smaller flocks in the same village may be combined together to form a single large village flock managed by a group of shepherds. There is no regulation of animal movement in Egypt and livestock move freely across the country [18].

### Study design

A cross-sectional study was conducted between February and July 2014 to estimate the seroprevalence of brucellosis in the sheep population and collect information on KAPs of shepherds towards brucellosis in the Kafrelsheikh district, Egypt.

### Sampling

The Kafrelsheikh district has 10 main villages where sheep are managed in flocks by a number of shepherds. Our target population was all the sheep (n = 24,000) in the 10 villages. Each of these villages was assumed to have a similar flock size. The total number of sheep was calculated using a 2010 census and individual sheep were the primary sampling units. The sample size was estimated using Win episcope 2.0 with an expected prevalence of 15 and a 5 % accepted error being 196 animals. We increased this sample size to 270 sheep [9]. This number was divided equally across the main 10 villages. In each of the villages, the total desired sample of 27 sheep was equally divided between the present flocks. Within each flock, sheep were selected by simple random sampling. A total of 273 serum samples were collected from 28 flocks in the 10 villages.

### Serological testing

Serum was extracted from whole blood by centrifugation at 3000 rpm for 15 min at 4 °C and stored at −20 °C until examined. The Rose Bengal Plate Test (RBPT) was conducted according to manufacturer’s manual (Prionics AG, Schlieren-Zurich, Switzerland) and samples positive by RBPT were subsequently tested by complement fixation test (CFT). Antigen for the CFT was obtained from the NVSL/DBL, USDA, USA. Complement and hemolysin were prepared and preserved according to Alton et al. [19] and were titrated according to Hennager [20]. Sheep erythrocytes were collected on Alsever’s solution from an adult healthy ram serologically negative for brucellosis and standardized to 3 % suspension in veronal buffer saline. Results of the CFT were interpreted as positive at a cutoff point of ≥20 ICFTU/ml [19].

### Epidemiology

The apparent seroprevalence (AP) of brucellosis was estimated as follows [21]:

$${\text{AP}} = ({\text{Number of animals seropositive to both RBPT and CFT}}/{\text{Number of examined animals}}) \times 100$$

The true seroprevalence (TP) of brucellosis was estimated as follows [21]:

$${\text{TP}} = {\text{AP}} + {\text{Se}} - 1/{\text{Se}} + {\text{Sp}} - 1$$

where TP is the true seroprevalence, Se is the in series combined sensitivity of both of RBPT and CFT (78 %) and Sp is the in series combined specificity of both RBPT and CFT (99 %) [9].

The confidence interval (CI) for the TP was obtained as follows [21]:

$$CI = p \pm Z*\sqrt {\frac{p*(1 - p)}{n}}$$

The village flock true seroprevalence (VFTP) for each of the 10 studied villages was calculated as VFTP = (Village flock AP + Sp − 1)/(Se + Sp − 1). The proportion of villages, which had at least one seropositive sheep after accounting for the village flock combined Se (VFCSe) and Sp (VFCSp) of serological tests was calculated as described by Hegazy et al. [9]. The VFCSe and VFCSp values used were 0.93 and 0.76 respectively [9].

### Questionnaire

Data concerning shepherd KAPs was collected using a structured questionnaire, developed in English and translated into Arabic. The questionnaire was piloted in one village, with three shepherds interviewed and the questionnaire subsequently revised. After revision, the questionnaire was then administered to all shepherds (n = 26).

The awareness of shepherds regarding brucellosis was investigated through the use of open questions concerning the main diseases causing abortion in sheep, whether or not these diseases affect humans, the main signs of the disease and potential routes of transmission to humans. Attitudes and practices relating to brucellosis were assessed by asking the shepherds questions regarding the use of hygienic measures in handling aborted material or recently aborted sheep and the role of veterinarians in such cases. The level of collaboration with the national control programme was investigated through questions regarding the number of visits undertaken by the General Organization of Veterinary Services campaigns annually to collect blood samples for serological examination for brucellosis. The questionnaire is shown in Additional file 1.

### Ethical approval

Ethical approval was obtained from the Committee of Research, Publication and Ethics of the Faculty of Veterinary Medicine, Kafrelsheikh University. All procedures were explained to flock owners and owners’ informed verbal consents were obtained.

## Results

### Seroprevalence

A total of 273 serum samples were collected from 28 sheep flocks in the 10 villages. The sera were examined by RBPT with agglutination recorded in 47 samples (17.95 %). Positive RBPT serum samples were confirmed by CFT with 16.48 % (45/273) of the samples being positive for both tests (AP). The TP was estimated at 20 % (95 % CI 15.3–24.7 %).

**Fig. 1:**
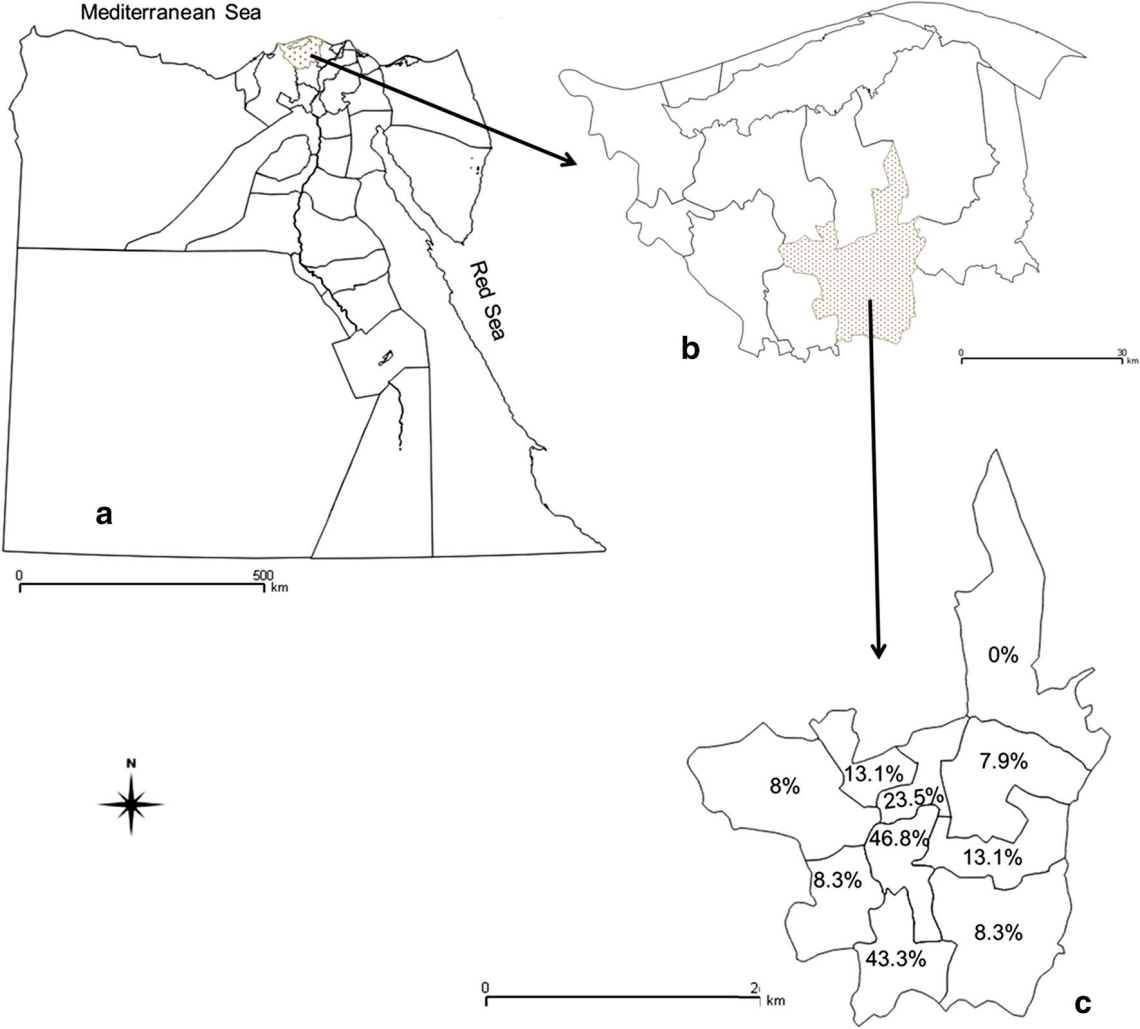
Map of Egypt showing **a** the administrative governorates: the dotted governorate is the Kafrelsheikh governorate (study area), **b** the administrative ten districts of Kafrelsheikh governorate, **c** Map for the ten villages of Kafrelsheikh district showing the village flock prevalence for each village

The prevalence of villages with at least one seropositive sheep for brucellosis after adjusting for the VFCSe and VFCSp was estimated at 95.5 % (95 % CI 92.2–100 %) with 9 out of 10 villages having at least one sheep that tested positive. The village flock prevalence ranged from 0 to 46.8 % (Fig. 1).

### Shepherds’ KAPs

Out of 26 shepherds who responded to the questionnaire, 16 (61 %) declared that brucellosis alone was the main causative agent of abortion in their flocks, while 4 (15 %) stated that Rift Valley Fever virus in combination with *Brucella* spp. were the main causative agents of abortion in their flocks. Six shepherds (23 %) did not give an answer to this question. The shepherds (n = 20), who answered the questions, believed that humans could be infected by *Brucella* spp. while assisting aborting ewes and considered this as the only route for disease transmission to humans. Five of 16 shepherds (31.3 %) described fever as a sign of human brucellosis while the rest were not aware of any signs of the disease in humans.

Out of 18 shepherds, 10 (55.5 %) fed the aborted materials to their dogs while 5 (27.8 %) throw aborted materials into the water canals and only 3 (16.7 %) bury aborted materials. Out of 21 shepherds, 15 (71.4 %) keep aborted animals in their flocks for further breeding seasons. Five shepherds (23.8 %) would sell these animals and only one shepherd said that he slaughtered them. Two of the shepherds, who would keep aborted animals in the flock, reported having suffered from fever and being diagnosed with brucellosis. Assisted parturition, not wearing protective gloves or masks when assisting with the parturition, slaughtering sheep and eating meat of slaughtered sheep were practiced by all shepherds interviewed.

Although all shepherds interviewed were aware of the official test and slaughter program for brucellosis control in Egypt, none of them had ever had their flocks tested by the veterinary authorities. Only one shepherd out of 19 (5.3 %) said that he might call the veterinarian, official or private, for advice regarding aborted animals.

## Discussion

Sheep are considered the primary source of *Brucella mellitensis,* which is the most pathogenic *Brucella* sp. in humans and the predominant strain circulating in Middle East, including Egypt [22, 23]. Recent non-governmental studies indicate that brucellosis is highly endemic in ruminants in Egypt, though large discrepancies in seroprevalence exist between peer-reviewed published studies and those reported by the government [9, 12].

The seroprevalence of brucellosis in sheep in the study area was estimated at 20 % (95 % CI 15.3–24.7 %). Official Egyptian government figures nationwide for *Brucella* seroprevalence in sheep between 1999 and 2011 range from 0.5 to 2.5 % [15]. The seroprevalence in this study is slightly higher than that reported by Hegazy et al. [13]. This study agrees with Hegazy et al. [13] stating that brucellosis is endemic in Egypt with a high seroprevalence (around 15 %) despite the current national control programme. This may be due to poor availability of resources, a lack of compliance among livestock owners and the structure of the local production systems. To our knowledge the proportion of villages with at least one sheep seropositive for brucellosis reported in the current study area is the highest ever reported.

Results from the KAPs survey show that official testing and culling had apparently never been conducted and the majority of shepherds tended to keep aborted animals within their flocks—both of these factors potentially responsible for the persistently high *Brucella* seroprevalence in the study area [13]. Free movement of flocks, lack of livestock identification, open livestock markets, unhygienic parturition measures and the throwing of aborted material into water canals are also significant in the transmission and persistence of the disease.

Results from the shepherd KAPs survey showed that most of the participant shepherds had good awareness of ovine brucellosis. Farmers participating in another study in Egypt also showed similarly high levels of awareness [12]. The high endemicity of livestock brucellosis in Egypt is likely to have increased public awareness, particularly among livestock owners and those working closely with livestock. Furthermore, the high economic impact of the disease and risks of human infection may have also strengthened this knowledge.

All the shepherds who answered the questionnaire identified brucellosis as the main cause of abortion within their flocks and were aware of the risks of human infection. They were not however aware of any potential modes of transmission to humans other than direct contact with aborted ewes and aborted material. As a result of this lack of awareness, shepherds continue high-risk practices including home slaughter of sheep and subsequent meat preparation [24]. None of the participant shepherds drank sheep milk though, due to a lack of awareness about cross-species transmission, they still drink milk from goats, even though their flocks may be suffering abortions in both sheep and goats.

Despite their knowledge of human brucellosis, only a few shepherds described fever as a sign of the disease. A lack of awareness about the signs of the disease may cause the seriousness of the disease to be underestimated, with infected shepherds not seeking immediate medical attention and thus exposing themselves to more severe complications of the disease. This underestimation of disease severity may also play a role in the shepherd’s ignorance regarding high-risk practices such as assisting parturition or handling of aborted material from ewes without gloves or masks [25]. This lack of awareness concerning signs of human brucellosis and modes of transmission may be attributed to inadequate communication by the public health authorities, shortage of awareness campaigns usually associated with the underreporting of disease and inadequate surveillance [8].

Most farmers and shepherds infected with brucellosis do not share information about their illness with the public health authorities, veterinarians or even their co-workers for fear of the economic losses caused by governmental tracing and culling of their livestock [12]. Infected shepherds thus fail to add to the knowledge about brucellosis in their community, facilitate underreporting and hinder control programmes. Pappas et al. [6] reported that among brucellosis patients’ in Greece this attitude of not allowing veterinary investigation, for fear of an adverse effect on their herd, was associated with an increased incidence of human infections.

The majority of participants reported that they fed aborted fetuses to their dogs and this practice may also increase transmission and persistence of infection in the flock. Dogs play a role in mechanical transmission of the infection when they drag aborted material across the ground [3]. Some shepherds also throw aborted material into water canals used by sheep and other livestock for drinking or bathing. Shepherds, farmers and other village residents come into contact with this water though daily routines such as bathing, irrigation of fields, washing of utensils, fishing and other activities. The practice of discarding aborted material into watercourses is a likely cause of water contamination and increases the risk of disease transmission to human and livestock populations in the region [26].

Most shepherds kept aborted animals in their flocks, while a few reported they might sell them at market. Only one shepherd mentioned slaughtering as a possible course of action. This is in contrast with Holt et al. [12], who found that most farmers preferred to sell infected animals in the market (80.4 %) or directly to the butcher (50.5 %) and none would keep such animals. These differences in attitude may be attributed to differences in knowledge of the disease, with farmers showing a high degree of brucellosis awareness and accurate knowledge of the disease, its transmission and its effects when compared to shepherds [12]. This knowledge may help in guiding farmers toward selling infected animals rather than keeping them and thus exposing their households to the risk of infection. Shepherds in this study however lacked knowledge regarding the public health risk of keeping infected animals within their flock. Another possibility is the economic benefit, as it seems that keeping mature ewe for production of offspring is more profitable for shepherds than selling them in the market as this may decrease the production capacity of their flocks.

Only one shepherd stated that he had asked for advice from a veterinarian in an abortion case, while the remaining shepherds claimed they never consulted the veterinarian in cases of abortion. Shepherd attitudes were shown to be very different from farmer attitudes in a village in Menufiya Governorate, Egypt, (and elsewhere) as most farmers preferred to consult a veterinarians, while shepherd consider dystocia management as a required skill for their profession and were reluctant to contact veterinarians [12, 27]. This lack of contact with veterinarians reduced their knowledge of risks and modes of infection transmission for brucellosis, as shown in this study. Consulting a veterinarian may be an important factor in improving awareness regarding brucellosis risks for both shepherds and farmers [27].

None of the shepherds were willing to notify the veterinary authorities in cases of abortion. All shepherds stated that no sampling by the veterinary authorities had ever been undertaken in their flocks for brucellosis (or any other disease) though they were aware of the official test and slaughter policy. In their opinion, this policy is economically unfair and potentially devastating to their flocks. Shepherds in this study and farmers in another study shared their dissatisfaction with the official brucellosis control programme in Egypt, particularly the system of compensation [12]. Based on farmers and veterinarians opinions, the official compensation monetary value for sheep was estimated to be less than 20 % of the actual market value [12]. As a result shepherds and farmers usually seek alternative economic choices, such as selling animals to butchers, or in the market, or simply keeping the animal for continued breeding purposes, as reported by many shepherds in this study. These factors are likely to be an on-going problem, hindering effective brucellosis control in Egypt.

## Conclusions

The findings of this study demonstrate that brucellosis is widespread in sheep of the Kafrelsheikh district, Egypt, despite a national control programme operating in the region since 1985. This study recommends control measures to decrease the public health risks associated with brucellosis in Egypt and reduce seroprevalence in sheep.

The results of this study show that eliminating aborted sheep from a flock is an economically favorable way of potentially reducing *Brucella* seroprevalence and it is expected that this information will prove useful in changing the reluctance of shepherds to slaughter apparently healthy animals. Educational campaigns to increase awareness of brucellosis among shepherds are urgently required. Such campaigns must also highlight the importance of disposing placentas and aborted fetuses appropriately and avoiding the high risks associated with (1) throwing aborted materials into water canals (2) home slaughtering of aborted animals and unhygienic handling of their meat (3) lack of protective measures during birth-aid with aborted animals as wearing gloves and using of antiseptics.

## Additional file

**Additional file 1.** Questionnaire used to collect shepherds’ knowledge, attitudes and practices on ovine brucellosis.

## Abbreviations

AP: apparent seroprevalence
CFT: complement fixation test
CI: confidence interval
KAPs: knowledge, attitudes and practices
RBPT: rose bengal plate test
TP: true seroprevalence
ICFTU: International Complement Fixation Test Units
NVSL/DBL, USDA: National Veterinary Services Laboratories/Diagnostic Bacteriology Laboratory, United States department of Agriculture
VFTP: village flock true seroprevalence
VFCSe: village flock combined sensitivity
VFCSp: village flock combined specificity
