# Supplementary material for: Seroprevalence and “Knowledge, Attitudes and Practices” (KAPs) survey of endemic ovine brucellosis in Egypt
Source: Acta Vet Scand. 2016 Jan 7;58:1. doi: 10.1186/s13028-015-0183-2 (PMC4704395; doi:10.1186/s13028-015-0183-2)
Supplement: Supplementary file 1 — Additional file 1. Questionnaire used to collect shepherds’ knowledge, attitudes and practices on ovine brucellosis. [file 13028_2015_183_MOESM1_ESM.pdf]

Date: **Location ID:** **Flock ID:**

Questionnaire used to collect shepherds' knowledge, attitudes and practices on ovine brucellosis

Our aim for conducting this questionnaire is to investigate causes of abortion and its potential public health implications. This is to find suitable ways for preventing such diseases introduction and transmission within sheep flock, hence increasing sheep productivity and avoiding its potential occupational health risks. All data obtained in this questionnaire is confidential.

(A) Identification:

1-Shepherd's name (optional): ……………………………………………………………

2- Shepherd's phone (optional):...………………………………………………………..

3-Address:……………………………………………………………………………….

(B) Flock information and management:

4-Flock size (sheep):

< 50 □ > 50 □

5- Do you have co-worker for raising your sheep flock?

Yes □ No □

If Yes:

6- Number: ☐

7– Do you have other animal species mixed with your flock?

Yes □ No □

- If Yes:

8- What are these species?

Cattle ☐ Buffaloes ☐

Equines ☐ Goats ☐

9- Do you mix your flock with household sheep or goats belong to farmers which join the flock for specific period?

Yes ☐ No ☐

10-Flock type:

Fixed ☐ Mobile (free grazing) ☐

If Mobile:

11- Places to which your flock moves over the year:

Only in Kafrelsheikh ☐ Kafrelsheikh /Gharbia ☐ Kafrelsheikh /Other ☐

(C) Abortions in the flock:

12- Do abortions occur in your flock:

Yes ☐ No ☐

If Yes:

13- How many of breeding ewes aborted last year? ☐

14- Do you call the veterinarian after a case of abortion?

Yes ☐ No ☐

(D) Shepherds' knowledge and practices:

15- Do you eat meat from your sheep flock?

Yes ☐ No ☐

16- Do you personally slaughter sheep from your flock?

Yes ☐ No ☐

17- Do you involve in delivery of pregnant ewe?

Yes ☐ No ☐

If Yes:

18- How do you involve?

Birth-aid ☐ Assistance to veterinarians ☐ Giving IU medication ☐

19- Do you use gloves?

Yes ☐ No ☐

20- Do you involve in delivery of pregnant ewe with abortion?

Yes ☐ No ☐

If Yes:

21- How do you involve?

Birth-aid ☐ Assistance to veterinarians ☐ Giving IU medication ☐

22- Do you use gloves?

Yes ☐ No ☐

23- What do you do with aborted feti?

Dumped on the banks of canals ☐ Burial ☐ Burning ☐ Fed to dogs ☐

24- What do you do with a ewe with abortion?

Sell it ☐ Keep it ☐ Slaughter it ☐ Segregate it ☐

25- In your opinion what are the diseases causes abortions in sheep?

………………………………………………………………………………………

26- Do you think that sheep can infect humans with any of these diseases?

Yes ☐ No ☐ Don't Know☐

If Yes:

27- What are these diseases?

……………………………………………………………………………………….

28- What are the symptoms of these diseases?

……………………………………………………………………………………….

29- What are the potential routes of their transmission?

……………………………………………………………………………………….

<u>(E) Veterinary authorities' disease control:</u>

30- What are the actions taken by the veterinary authorities when Brucellosis is discovered in a flock?

Slaughtering of the reactors ☐ Vaccination of the flock ☐ Both ☐ Don't Know☐

31- Did the official veterinary authorities take any samples from your flock for testing against Brucellosis last year?

Yes ☐ No ☐

- If Yes:

32- What are the types of samples taken?

Blood ☐ Stool ☐ Vaginal discharges ☐ Others ☐

33- How many times per year does sampling occur?

Once /year ☐ Twice/year ☐ Three or more/year ☐

(F) Cases of Brucellosis among Shepherds:

34- Did you ever diagnosed with Brucellosis?

Yes ☐ No ☐

If Yes:

35- How the illness was diagnosed:

Private lab ☐ Hospital ☐

36- Location of medical care:

Household ☐ Hospital ☐

37- Did you involved in delivery of pregnant ewe with abortion before illness:

Yes ☐ No ☐
